# Early heart development: examining the dynamics of function-form emergence

**DOI:** 10.1042/BST20230546

**Published:** 2024-07-09

**Authors:** Noémie Combémorel, Natasha Cavell, Richard C.V. Tyser

**Affiliations:** Cambridge Stem Cell Institute, University of Cambridge, Jeffrey Cheah Biomedical Centre, Cambridge CB2 0AW, U.K.

**Keywords:** cardiovascular physiology, cardiovascular progenitor cells, developmental biology, embryogenesis, heart

## Abstract

During early embryonic development, the heart undergoes a remarkable and complex transformation, acquiring its iconic four-chamber structure whilst concomitantly contracting to maintain its essential function. The emergence of cardiac form and function involves intricate interplays between molecular, cellular, and biomechanical events, unfolding with precision in both space and time. The dynamic morphological remodelling of the developing heart renders it particularly vulnerable to congenital defects, with heart malformations being the most common type of congenital birth defect (∼35% of all congenital birth defects). This mini-review aims to give an overview of the morphogenetic processes which govern early heart formation as well as the dynamics and mechanisms of early cardiac function. Moreover, we aim to highlight some of the interplay between these two processes and discuss how recent findings and emerging techniques/models offer promising avenues for future exploration. In summary, the developing heart is an exciting model to gain fundamental insight into the dynamic relationship between form and function, which will augment our understanding of cardiac congenital defects and provide a blueprint for potential therapeutic strategies to treat disease.

## Introduction

The adult human heart has a distinct form and function, beating on average 3.5 billion times during our lifetime, however during early heart development cardiac form and function are highly dynamic. The heart is the first organ to form and function in the embryo proper, providing sufficient nutrients and oxygen to enable embryonic growth and development. In the human, the first morphologically recognisable heart structure forms at ∼3 weeks after conception and undergoes a complex morphogenetic rearrangement to form the four-chambered heart by around week seven of gestation [1]. During this period the heart begins to contract, with heart rate increasing to ∼180 bpm by 10 weeks post conception. In this review, we refer to ‘function’ as the ability of cardiomyocytes to contract, and to ‘form’ as the morphological characteristics of the cardiac tissue, which is underpinned by tissue remodelling and cellular events such as differentiation. Studying the dynamics of human form and function emergence is difficult given the exceptional rarity of human fetal donations at these early stages of development. Therefore, model organisms such as chick, zebrafish and mouse are fundamental to explore these processes, although cardiac physiology and the nature of these morphogenetic process vary between species [2]. This review will focus predominantly on the earliest stages of heart development, when form and function first emerge.

## Emergence of form

**Figure 1.**
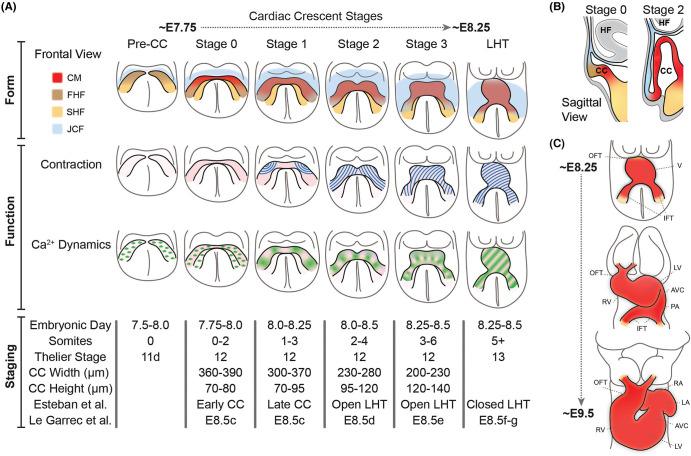
Emergence of form and function during early heart development. (**A**) Schematic presenting the parallel emergence of form and function during cardiac crescent development, highlighting cardiac crescent (CC) morphology and heart fields (as identified in Tyser et al. [3]), contraction (blue lines) and Ca^2+^ dynamics. Green stripes represent Ca^2+^ transients whilst green dots represent single-cell Ca^2+^ oscillations. FHF, first heart field; SHF, second heart field; CM, cardiomyocyte; JCF, juxta cardiac field; LHT, linear heart tube; Pre-CC, pre-cardiac crescent. Table describes cardiac crescent staging systems, including two other recent systems specific to the heart [4,5]. (**B**) Schematic sagittal sections through the cardiac crescent at two different developmental stages depicting buckling of the cardiac crescent and lumen formation. CC, cardiac crescent; HF, headfold. (**C**) Schematics showing the morphology changes during the transition from linear heart tube to looped heart from a frontal view. OFT, outflow tract; V, ventricle; IFT, inflow tract; RV, right ventricle; LV, left ventricle; AVC, atrioventricular canal; PA, primitive atrium; RA, right atria; LA, left atria.

Cardiac morphogenesis starts with formation of the cardiac crescent, the first morphologically recognisable heart structure in the developing embryo (Figure 1A). The cardiac crescent is an arch of differentiating cardiomyocytes in the rostral portion of the embryo, which forms at around embryonic day (E)8 in the mouse (∼day 20 post fertilisation in the human) [6]. It is derived from mesoderm, which arises during gastrulation, as pluripotent stem cells of the epiblast undergo an epithelial-to-mesenchymal transition through the caudally positioned primitive streak, at ∼E6.25 (∼14–15 days post fertilisation in human) [7]. This mesoderm migrates collectively as a sheet of cells away from the primitive streak and towards the rostral region of the embryo as bilateral wings. During this migration, the mesoderm undergoes a process of differentiation, forming distinct populations of cardiac progenitors with specific cell fates [8–11]. As the bilateral wings begin to meet at the midline, a rapid first wave of cardiac progenitor differentiation occurs in a region termed the first heart field, giving rise to the initial arc of immature contractile cardiomyocytes in the cardiac crescent (Figure 1A). A wider domain of cardiac progenitors, dorsal and medial to the cardiac crescent, termed the second heart field, remains proliferative and continues to add to the heart over subsequent development [12]. Our ability to anatomically and molecularly define cardiac progenitors has increased with the aid of new imaging approaches and single-cell RNA sequencing, revealing an unexpected complexity and novel progenitor types [10,13–17].

The cardiac crescent initially is a layer of cells sandwiched between the ventral endoderm and dorsal ectoderm (Figure 1B). As development proceeds, rostral folding positions the forming heart beneath the emerging headfolds and above the foregut pocket, leading to a buckling of the cardiac crescent and formation of a lumen encasing endocardial progenitors (Figure 1B). During subsequent development, the cardiac crescent increases in height and decreases in width, prior to fusing at the midline to form the linear heart tube (LHT). This morphogenesis occurs with limited progenitor differentiation [18]. The final stage in the progression from cardiac crescent to LHT is the closure of the dorsal myocardium to form a closed tube. This tube is segregated into distinct morphologically defined regions, including the outflow tract, ventricle primordium, atria primordium and the inflow tract, that will later give rise to specific domains of the heart (Figure 1C) [19,20]. This transition from early cardiac crescent to LHT occurs in the mouse over a period of ∼12 h. To precisely assess this and gain insight into the emergence of cardiac form, staging systems which accurately represent these dynamic changes are essential [21]. A recent high-fidelity morphological quantification of murine heart tube formation showed that parameters correlating with cardiac crescent height-to-width ratio are the best classifiers of stage during the transition from cardiac crescent to LHT [4]. It also highlighted specific regions of the emerging heart with increased inter-individual variability, such as the dorsal borders of the myocardium or the inflow tract, regions that are highly dynamic and transient during early heart development.

Following its formation, the LHT begins to loop rightwards while simultaneously elongating cranially and caudally by addition of cells, initiating the formation of the functional four-chambered heart [5,22]. Initiation of the looping process represents the first morphological left/right (L/R) symmetry breaking in the embryo, and is preceded by L/R differential gene expression in the cardiac crescent [23,24]. During looping, the right and left cardiac chambers expand, in a phenomenon termed ballooning [19]. The right ventricle bulges ventrally and moves caudally to its final position on the right as the tube axis tilts, whilst the outflow tract extends (Figure 1C). Quantitative analyses and computer modelling of cardiac looping is providing unique insight into how asymmetric organ morphogenesis occurs [5,25]. One such model shows that cardiac looping can occur due to the LHT buckling between two fixed poles. This is caused by asymmetries at the poles which generate opposite deformations [24,26]. Thus, looping morphogenesis is caused by complex mechanical forces rooted in cellular processes such as differentiation, growth, extracellular matrix breakdown and cellular chirality [5,26–28]. Recent work showed that genetic perturbation in cardiac progenitors can cause torsion defaults, with some leading to a congenital defect termed crisscross heart, revealing how progenitor cell dynamics influence mechanical forces to impact tissue-scale organisation [26,29].

Over subsequent development the chambers of the heart mature, with the formation of ridges called trabeculae, which protrude into the lumen of the ventricles before undergoing compaction, resulting in a thick layer of myocardium [30,31]. Atrial and ventricular septation along with mitral and tricuspid valve formation allow for the establishment of a dual pulmonary-systemic circulatory system with unidirectional blood flow [32]. Mechanical stimuli, cellular contractility and tension heterogeneity have been shown to be important regulators of these later morphogenetic processes, highlighting the interplay between function and morphogenesis throughout cardiac development [33–35]. In summary, formation of the heart represents a highly dynamic remodelling process, involving the complex coordination of cellular events, such as differentiation, proliferation and migration, whilst undergoing constant contraction and relaxation.

## Emergence of function

**Figure 2.**
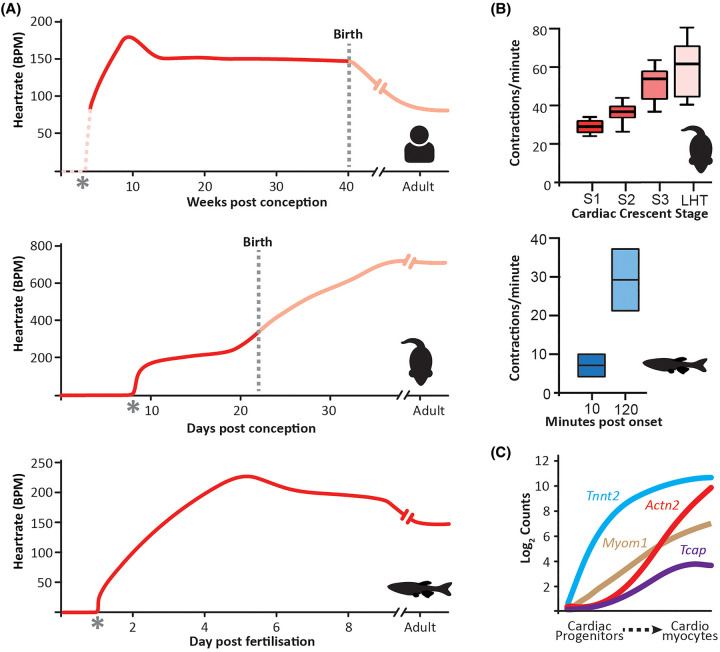
Functional dynamics during heart development. (**A**) Heart rate dynamics during the course of development in different species (Human, Mouse, Zebrafish), highlighting the early onset of function and species-dependent variation in heart rate [21,37–44]. Asterisks mark the morphological emergence of the heart, dashed pink line highlights the unknown dynamics of heart rate emergence in the human. * (**B**) Bar graphs show the rapid increase in function in the mouse (top panel) [37] and zebrafish (bottom panel, ∼20 h post fertilisation) [41]. (**C**) Expression levels of sarcomere-related genes plotted against the diffusion pseudotime of cardiac progenitor to cardiomyocyte differentiation during mouse cardiac crescent formation [3]. Lines represent the quadratic local linear fit of the expression levels as a function of pseudotime. *Tnnt2*, cardiac muscle troponin T; *Myom1*, myomesin; *Tcap*, telethonin; *Actn2*, α-Actinin 2.

The emergence of cardiac function has been an area of interest for centuries, with early embryonic contractile activity first observed directly through the microscope in the 1920s by the pioneering female scientist Florence Sabin, whilst studying blood development in the chick [36]. Model systems (chick, zebrafish, mouse) are fundamental when exploring the emergence of function at the tissue scale level, and whilst there is conservation in the fundamental processes underlying cardiac function, significant differences in contractile rate and cardiac electrophysiology exist between species (Figure 2A).

In the emerging embryonic mouse heart, the onset of contractility occurs in tandem with formation of the cardiac crescent (Figure 1A). Initial contractions are first detected in the left and right lateral regions of the early cardiac crescent, ∼E8.0, at ∼30 contractions per minute (Figures 1A and 2B) [37]. As the cardiac crescent transitions to the LHT, the rate of contraction increases to more than 60 contractions per minute, thus doubling within an 8 to 10-h time period. During subsequent development, the embryonic heart rate rises to ∼200 bpm at E12.5, before increasing to over 500 bpm in the adult mouse [38]. Zebrafish have an adult heart rate more comparable to the human at ∼150 bpm, although their cardiac morphology is significantly different, with a two-chambered heart and a more rudimentary conduction system [39,40]. In Zebrafish, it has been shown that 10 min post the onset (mpo) of cardiac function, the heart is contracting at ∼7 bpm, rising to ∼30 bpm at 120 mpo (Figure 2B) [41], before reaching a maximum heart rate of ∼232 bpm in the juvenile fish (∼5 days post fertilisation), and then decreasing in the adult. Whilst the initiation of contraction cannot be assessed precisely in the human, the human foetal heart rate also varies during pregnancy with a steady increase up to the end of the ninth week of gestation, reaching a peak of ∼170 bpm followed by a gradual decrease until the end of the first trimester, after which the heart rate plateaus at ∼150 bpm, before decreasing after birth (Figure 2A) [42,44]. These rate changes are underpinned by the cellular composition of the developing heart, the changes in cellular electrophysiology and the mechanism of action potential initiation and propagation [3,45,46].

For the heart to start contracting, cardiomyocytes require both sarcomeres, the functional force-generating unit, and the ability to dynamically regulate cytoplasmic calcium (Ca^2+^) concentrations. Sarcomeres are composed of overlapping actin-thin and myosin-thick filaments arranged in bundles called myofibrils [47]. Contractile proteins such as cardiac troponin T (cTnT), sarcomeric α-actinin (α-Actinin) and myomesin (Myom) are expressed within the early cardiac crescent prior to the onset of contraction, with sarcomere assembly being detected shortly after the co-expression of these proteins and coinciding with the initiation of contractile activity [21]. During the emergence of function, contractile proteins show different spatial localisation in the cardiac crescent. cTnT can be detected throughout the early cardiac crescent, whilst α-Actinin and Myom at the same stage are detected only in the lateral regions (Figure 2C) [18,48]. This difference likely represents the maturity of cardiomyocyte differentiation, with more ‘mature’ cardiomyocytes being localised in the lateral regions of the crescent, corresponding with where initial contractions are first optically observed. The development of single-cell sequencing and computational approaches has allowed for the temporal dynamics of sarcomere formation at the genetic level to be resolved in more detail. In the mouse, for example, *Tnnt2* (encoding cTnT) is expressed in cardiac progenitors, increasing as cardiomyocyte differentiation proceeds [3]. In contrast, genes such as *Actn2* (α-Actinin) and *Myom1* (Myomesin) are up-regulated slightly later and restricted to cardiomyocytes (Figure 2C). As well as being fundamental for contraction, sarcomere formation can also drive cardiomyocyte maturation, with α-Actinin regulating both structural organisation as well as transcriptional maturation of cardiomyocytes through Serum response factor signalling [49], highlighting the close interplay between form and function during heart development. The onset of contraction will also mark the emergence of dynamic mechanical forces which will be applied to the developing heart such as stretch, sheer stress, flow and stiffness. These forces have been shown to impact form at both the tissue and cellular levels through the activation of different signalling pathways and the regulation of chromatin organisation [50–56].

Cardiomyocyte contraction is underpinned by electrical activity which regulates the concentration of cytoplasmic Ca^2+^ [57]. Elevated cytoplasmic Ca^2+^ binds to troponin, leading to a conformational change, enabling the sliding of the myosin and actin filaments and the shortening of sarcomere length, leading to force generation [58,59]. Removal of Ca^2+^ from the cytoplasm leads to relaxation. These changes in cytoplasmic Ca^2+^ concentration can be measured with organic dyes or transgenic reporter systems, thus providing a readout of function. Using these reporters, it has been shown across different species that rhythmic calcium dynamics and action potential activity can be detected prior to the onset of contraction in the emerging heart [41,48,60,61]. Once synchronised calcium transients have been established they propagate laterally across the cardiac crescent before transitioning to spread from the left inflow region of the LHT in a posterior-anterior direction (Figure 1A) [48]. As the heart loops, the initiation site of Ca^2+^ transient propagation transitions from the left to the right inflow region, highlighting a spatially distinct switch in cardiac pacemaking [62,63].

Prior to the onset of synchronised propagating waves, cardiac progenitors exhibit low-frequency single-cell spontaneous asynchronous Ca^2+^ oscillations [48]. These oscillations are independent and distinct from the synchronised Ca^2+^ transients which are initiated at the onset of contraction [41]. Thus, emergence of function represents a step change from slow single-cell oscillations to propagating tissue-scale transients, two separate phenomena. As well as regulating function, Ca^2+^ can also act as a second messenger in many signalling pathways. In the context of heart disease, multiple calcium-dependent signalling pathways such as CaMKII, calmodulin and calcineurin have been described to modulate gene expression [64]. In line with this, Ca^2+^ handling within early cardiac progenitors has been shown to promote cardiomyocyte differentiation and early cardiogenesis [21,48,65].

In mature cardiomyocytes, the mechanism by which electrical activity is coupled to Ca^2+^ dynamics is termed excitation contraction coupling (ECC) [66]. ECC requires a distinct set of channels, exchangers and pump proteins to trigger sarcoplasmic reticulum (SR) Ca^2+^ release and uptake [67]. During the emergence of function, ECC is not required, and knockout of ECC components results in the majority of transgenic mice hearts remaining contractile until ∼E11.5 [68,69]. The ability of emerging immature cardiomyocytes to spontaneously contract is lost during maturation, due to distinct transcriptional fate programs (i.e. ventricle, atria, pacemaker) and changes in cardiac electrophysiology [21,62,70–73]. During formation of the LHT, Ca^2+^ transient generation relies predominantly on plasmalemmal Ca^2+^ flux with limited SR Ca^2+^ contribution. At looping stages (E8.5), changes in cytoplasmic Ca^2+^ concentration are driven by a combination of both plasmalemmal Ca^2+^ influx and spontaneous SR Ca^2+^ release via ryanodine receptor (RyR) and inositol 3-phosphate receptor (IP_3_R) channels [21,48,73–76]. At the earlier cardiac crescent stages of development, RyR and IP_3_R inhibition do not inhibit Ca^2+^ transients, indicating that contraction is predominantly regulated by plasmalemmal Ca^2+^ influx, highlighting how Ca^2+^ handling evolves during heart development [48]. In summary, the emergence of function represents an adaptive process in which contractile rate is initiated and rapidly increases. This is underpinned by the formation and maturation of sarcomeres and by changes in cardiac electrophysiology.

## Conclusion/future perspectives

The embryonic heart represents a model system in which the emergence of form occurs in parallel with the onset and maturation of function. It therefore provides a unique model to explore how dynamic physiological and biophysical properties emerge and influence cell differentiation and tissue morphogenesis.

Investigating the emergence of form and function *ex vivo* in intact embryos is technically challenging due to the size, fragility and accessibility of tissue. However, this is becoming increasingly feasible thanks to technological developments in relation to imaging-based approaches and embryo culture. Recent papers have highlighted the ability to image, in combination with reporters, the emerging heart at single-cell resolution over extended periods [13,17,18]. This provides a window to examine the emergence of both form and function at the cellular level within intact embryos. Lightsheet microscopy has recently been used to image and track, at single-cell resolution, the migration of cardiac progenitors in the bilateral mesodermal wings during formation of the cardiac crescent [13]. This provided unique insight into how cardiac form emerges, highlighting how during mesodermal wing migration, cardiac progenitors do not maintain strict neighbour relationships and that cardiac progenitor subpopulations have distinct cellular behaviours. Migrating mesoderm cells also exhibit a variety of cell shape changes, which are determined by their spatial localisation in the embryo and regulated by different guidance, adhesion, cytoskeleton and matrix components [77–79]. In the early pre-gastrulating embryo, cell shape fluctuations and mechanical forces have been shown to regulate early lineage segregation [80]. It will therefore be interesting to explore how these distinct mechanical cues in the migrating mesoderm influence differentiation and lineage commitment. The ability to image and quantify heart development over extended time periods at the tissue-scale level in intact embryos will provide an opportunity to further understand how the biomechanical forces generated during mesoderm migration and cardiac crescent formation influence cell fate and tissue morphology. Additionally, it will be exciting to explore how the emergence of distinct morphologies influences local biomechanical forces and cell shapes.

Whilst examining cellular dynamics is possible in embryos prior to the onset of contraction, one of the major issues with characterising cellular dynamics and tissue remodelling in the developing heart is the motion artefact associated with the onset of contraction. Typically, pharmacological inhibitors have been used to prevent contraction, although these can impact other cellular dynamics and embryo viability. Optical-based gating strategies are now being developed which enable samples to be imaged without pharmacological inhibition [81]. These systems can remove motion artefacts by capturing the heart at the same point throughout the cardiac contraction cycle. This provides an exciting opportunity to study form whilst function is maintained. These algorithms are particularly challenging to implement in the early developing heart, when heart rate is rapidly increasing and cardiac morphology changing, but are possible with adaptive imaging systems. Combining these live-imaging approaches, gating algorithms and transgenic reporter systems, will provide novel insights into the cellular dynamics of the contracitle heart at the tissue-scale level.

The increased ability to characterise early heart development will enable us to decipher fundamental principles relating to the emergence of form and function, but also gain mechanistic insight into the potential interplay between these processes. At the molecular level, we can now examine transcriptional changes in single cells at high temporal resolution [9], enabling hypotheses to be generated and tested regarding the emergence of function, such as sarcomere formation (Figure 2C) or cardiomyocyte physiology. However, whilst these datasets allow us to consider potential mechanisms, it is fundamental for function to be assessed at the protein level. A challenge going forward will be to combine single-cell molecular insight with quantified measurements regarding cellular phenotype and physiological characteristics in functional tissue. The development of *ex vivo* and organoid culture systems will allow these potential mechanisms to be perturbed and quantified, in a more accessible manner [82–85]. Murine embryos have recently been cultured for up to 6 days from ∼E5.5, thus spanning emergence of the heart, including looping and chamber formation [86]. The rapid increase in *in vitro* human 3D models will enable us to explore mechanisms relating to the emergence of form and function, as it pertains to aspects of human heart development. Whilst these systems provide unique opportunities to explore the interplay between form and function, it is crucial to we have good references to validate them. New atlases characterising human heart development at the morphological and molecular level will provide invaluable insight when validating and benchmarking these *in vitro* models (Table 1) [1,87–98]. However, model systems from other species will remain fundamental, given the complex and dynamic nature of heart development and the spatially distinct cellular niches, which exist both within the heart and in between surrounding tissues.

**Table 1.** Single cell sequencing datasets characterising human heart development.

References	Year	Developmental stage	Sample type	Technologies
[87]	2019	4.5–5, 6.5 and 9 pcw	Heart	10x scRNA-seq, spatial transcriptomics
[88]	2023	4–6 pcw (CS12–16) (spatial CS13)	Whole Embryo	10x scRNA-seq, 10x Visium spatial transcriptomics
[89]	2023	∼5 and 7 pcw (CS16 and CS19)	Heart	10x spatial transcriptomics
[90]	2024	9–16 pcw (spatial 12/13 pcw)	Heart	10x scRNA-seq, MERFISH
[91]	2024	6–12 pcw	Heart	10x scRNA-seq, 10x Visium spatial transcriptomics
[92]	2024	4–20 pcw	Heart	10x scRNA-seq, 10x RNA/ATAC-seq Multiome, 10x Visium spatial transcriptomics
[93]	2024	4–20 pcw	Heart	10x scRNA-seq

Mouse datasets covering early heart development are collated in Sendra et al. Table 2 [9].

In summary, the emergence of form and function during early heart development represents a dynamic process of differentiation and tissue remodelling that occurs concurrently with the onset of contraction and changes in cardiac electrophysiology. Given the intertwined nature of its development, this organ is an exciting model system to explore how these individual phenomena emerge but also how they influence each other to impact subsequent development.

## Perspectives

- Formation of the heart and the onset of cardiac function occur during early embryogenesis. Over subsequent development cardiac form and function develop in parallel.
- Recent imaging and sequencing technologies have provided the ability to characterise the emergence of heart and the onset of function at single-cell resolution.
- This characterisation will enable novel mechanistic understanding of how cardiac form and function emerge, and augment our insight into how these two phenomena interact to regulate subsequent heart development.

## Open Access

Open access for this article was enabled by the participation of University of Cambridge in an all-inclusive *Read & Publish* agreement with Portland Press and the Biochemical Society under a transformative agreement with JISC.

## Abbreviations

ECC: excitation contraction coupling
LHT: linear heart tube
SR: sarcoplasmic reticulum
